# Supplementary material for: A new class of hybrid secretion system is employed in Pseudomonas amyloid biogenesis
Source: Nat Commun. 2017 Aug 15;8:263. doi: 10.1038/s41467-017-00361-6 (PMC5557850; doi:10.1038/s41467-017-00361-6)
Supplement: Supplementary file 1 — Supplementary Information [file 41467_2017_361_MOESM1_ESM.pdf]

SI GUIDE

File Name: Supplementary Information
Description: Supplementary Figures and Supplementary Tables.

Supplementary Data

File Name: Supplementary Data 1
Description: Primer sequences used for cloning and mutation construction.

File Name: Supplementary Data 2
Description: MS/MS sequencing of FapA peptides observed in whole cell lysates of Pseudomonas sp. UK4 expressing the fap-operon. The table lists identified peptides and how many times these were subjected to MS/MS sequencing. Two independent biological replicates were analyzed.

File Name: Supplementary Data 3
Description: MS/MS sequencing of FapB peptides observed in whole cell lysates of Pseudomonas sp. UK4 expressing the fap-operon. The table lists identified peptides and how many times these were subjected to MS/MS sequencing. Two independent biological replicates were analyzed.

File Name: Supplementary Data 4
Description: MS/MS sequencing of FapC peptides observed in whole cell lysates of Pseudomonas sp. UK4 expressing the fap-operon. The table lists identified peptides and how many times these were subjected to MS/MS sequencing. Two independent biological replicates were analyzed.

File Name: Supplementary Data 5
Description: MS/MS sequencing of FapD peptides observed in whole cell lysates of Pseudomonas sp. UK4 expressing the fap-operon. The table lists identified peptides and how many times these were subjected to MS/MS sequencing. Two independent biological replicates were analyzed.

File Name: Supplementary Data 6
Description: MS/MS sequencing of FapE peptides observed in whole cell lysates of Pseudomonas sp. UK4 expressing the fap-operon. The table lists identified peptides and how many times these were subjected to MS/MS sequencing. Two independent biological replicates were analyzed.

File Name: Supplementary Data 7
Description: MS/MS sequencing of FapF peptides observed in whole cell lysates of Pseudomonas sp. UK4 expressing the fap-operon. The table lists identified peptides and how many times these were subjected to MS/MS sequencing. Two independent biological replicates were analyzed.

File Name: Peer review File
Description:

Supplementary Table 1 Data collection, phasing and refinement statistics for all structures

	FapF$_{ß}$ SeMet	F103A	R157A
**Data collection**			
Space group	C121	C 1 2 1	P 1
Cell dimensions			
a, *b*, *c* (Å)	143.43, 124.57, 80.38	147.75, 125.82, 81.91	80.59, 125.71, 142.67
α, β, γ (°)	90.00, 96.32, 90.00	90.00, 96.68, 90.00	87.09, 84.70, 89.92
Wavelength	0.9798	0.9282	0.9795
Resolution (Å)	29.38-2.50 (2.64-2.50)	81.35-2.84 (2.89-2.84)	91.7-3.08 (3.13-3.08)
R_{pim}	0.059 (0.400)	0.149 (0.544)	0.103 (0.500)
$I / \sigma I$	10.8 (2.1)	7.2(1.6)	3.49 (1.2)
Completeness (%)	99.5	100(100)	99.02 (97.1)
Redundancy	3.4 (3.4)	13.4(13.4)	3.35 (3.4)
**Refinement**			
Resolution (Å)	2.50	2.840	3.08
No. reflections	164859	33293	94515
R_{work} / R_{free}	0.2211/0.2599	0.2316/0.2953	0.3208/0.3703
No. atoms			
Protein	6850	6524	23674
Ligand LDA/C8E/ ion	108 / 88 /3	64 /35/0	61/46/0
Water	229	13	0
B-factors			
Protein chain A/B/C	44.5	46.7	46.9
Ligand LDA/C8E/ ion	64.4/60.5/50.1	87.4/53.4/0	64.2/70.7/0
Water	43.2	38.8	-
R.m.s deviations			
Bond lengths (Å)	0.0132	0.0129	0.0114
Bond angles (°)	1.8042	1.7265	1.5465

*Data from a single crystal were used to solve the structure. *Values in parentheses are for highest-resolution shell.

Supplementary Table 2 Calculated collision cross sections from ion mobility mass spectrometry measurements

Charge state (+)	Conformation 1	Conformation 2	Conformation 3	Conformation 4
17	7035±203 Å^2			
18	6904±100 Å^2			
19	6863±81 Å^2			
20	6874±73 Å^2	7422±100 Å^2	8080±103 Å^2	
21	6978±164 Å^2	7530±109 Å^2	7971±117 Å^2	
22		7684±52 Å^2	8127±58 Å^2	
23		7798±44 Å^2	8252±63 Å^2	
24			8428±57 Å^2	
25			8508±100 Å^2	
26			8680±245 Å^2	
27				9073±135 Å^2
28				9074±95 Å^2
29				9142±65 Å^2
30				9248±100 Å^2

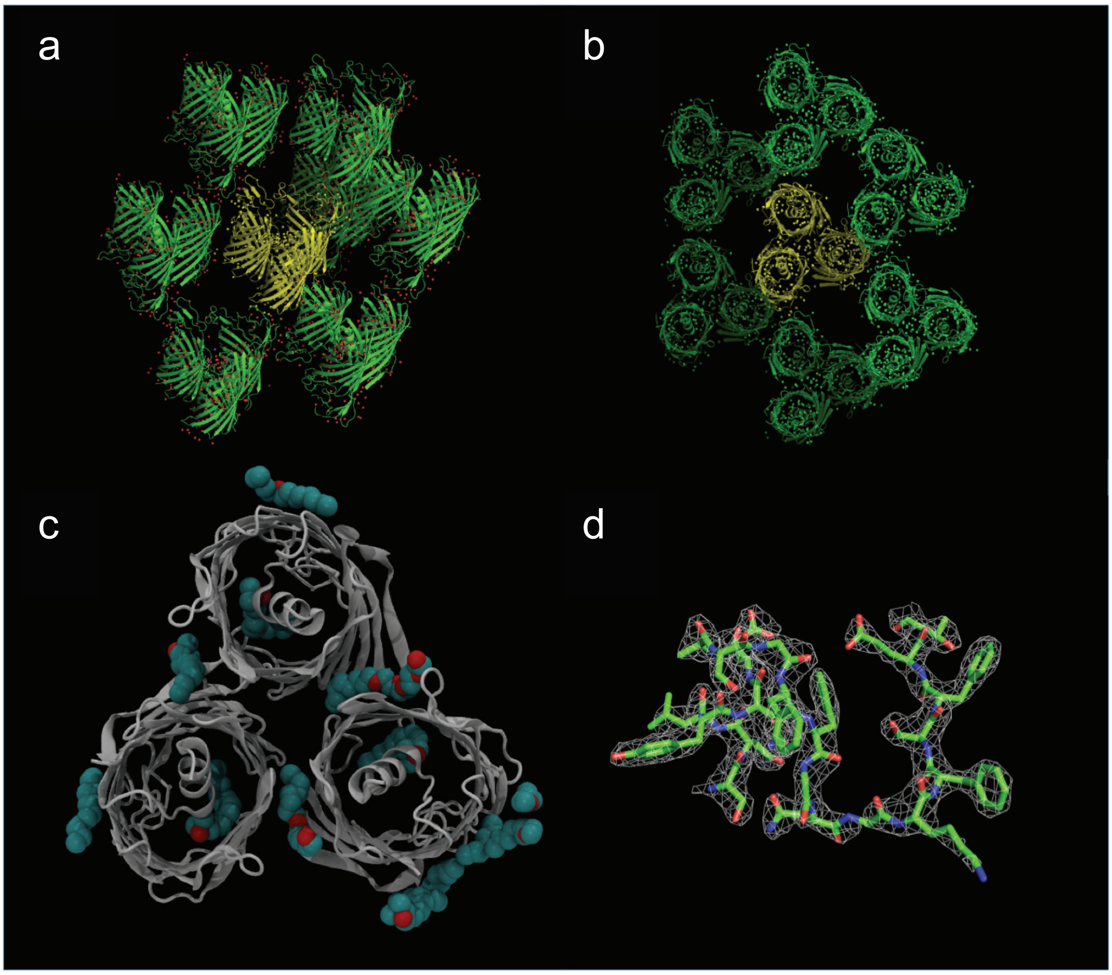

Supplementary Figure 1. Properties of wildtype FapFß crystal. (**a**) Sideview of crystal packing. The asymmetric unit is shown in yellow. (**b**) Top view of crystal packing. (**c**) View of FapFß indicating resolved detergent density attributed to LDAO and C8E4. (**d**) An example of the x-ray density quality.

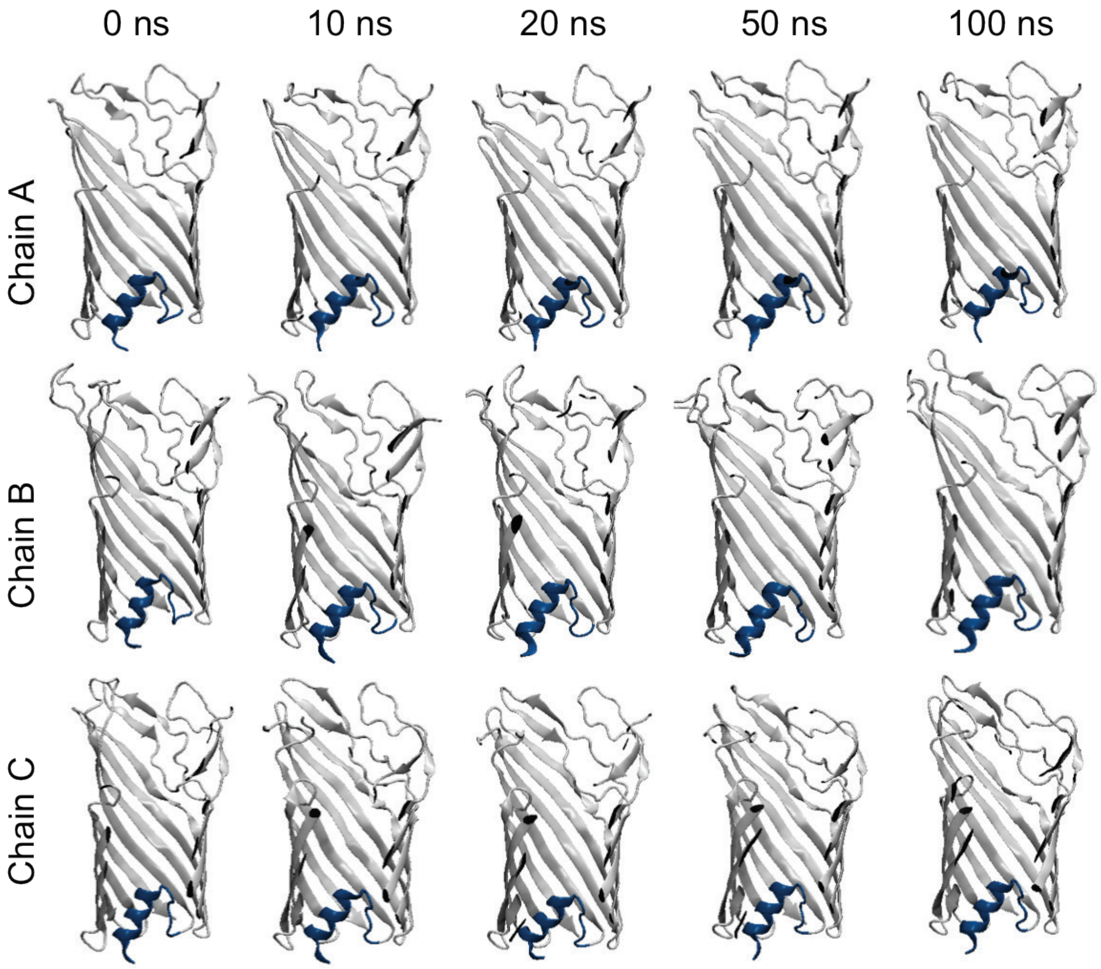

Supplementary Figure 2: The helical plug remains stable in each of the three subunits during molecular dynamics simulations in a simple lipid membrane (POPE:POPG ratio ~3:1). For clarity, the membrane and solvent (water and 0.15 mM NaCl) is not shown and the front of the barrel is cut away to reveal the helix plug and hairpin (blue). No ions are observed to pass through the constriction site during the 100 ns timescale of simulation.

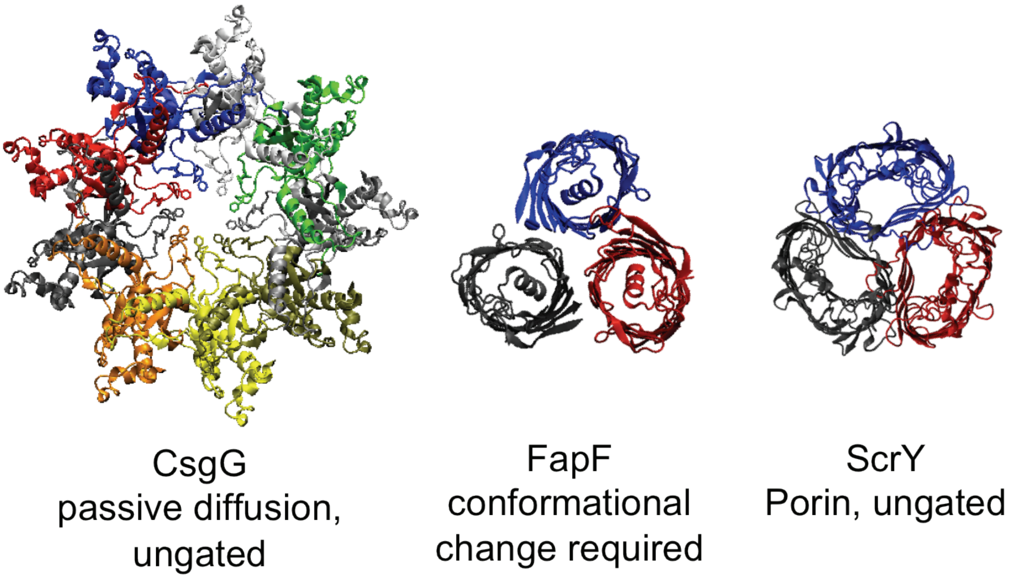

Supplementary Figure 3: A comparison of FapF to both CsgG, the amyloid secretion channel in *E. coli* (left; PDB id 4uv3), and the sucrose-specific porin ScrY (right; PDB id 1a0t). Chains are coloured individually. In CsgG the smallest constriction point is ~ 9 Å. In FapF the smallest constriction point is < 1.5 Å. In ScrY the smallest constriction is ~ 7 Å.

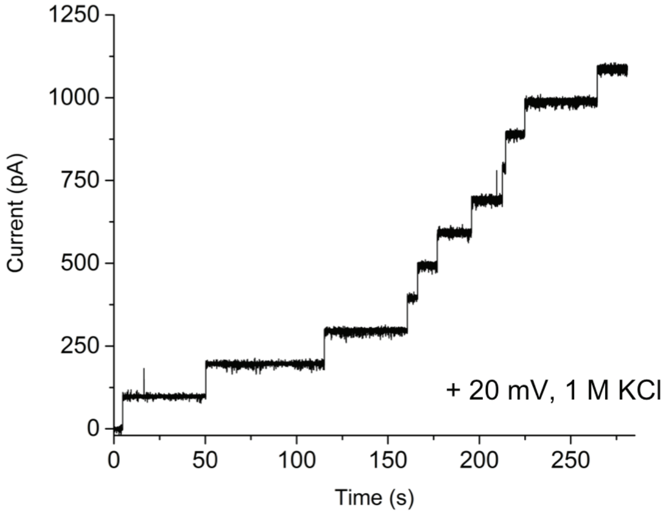

Supplementary Figure 4. Multiple full length FapF channels inserted under steady applied potential of +20 mV. The step-wise conductance for the full-length trimer in 1 M KCl, 20 mM Kpi (pH 7.0) is 4.95 ± 0.03 nS.

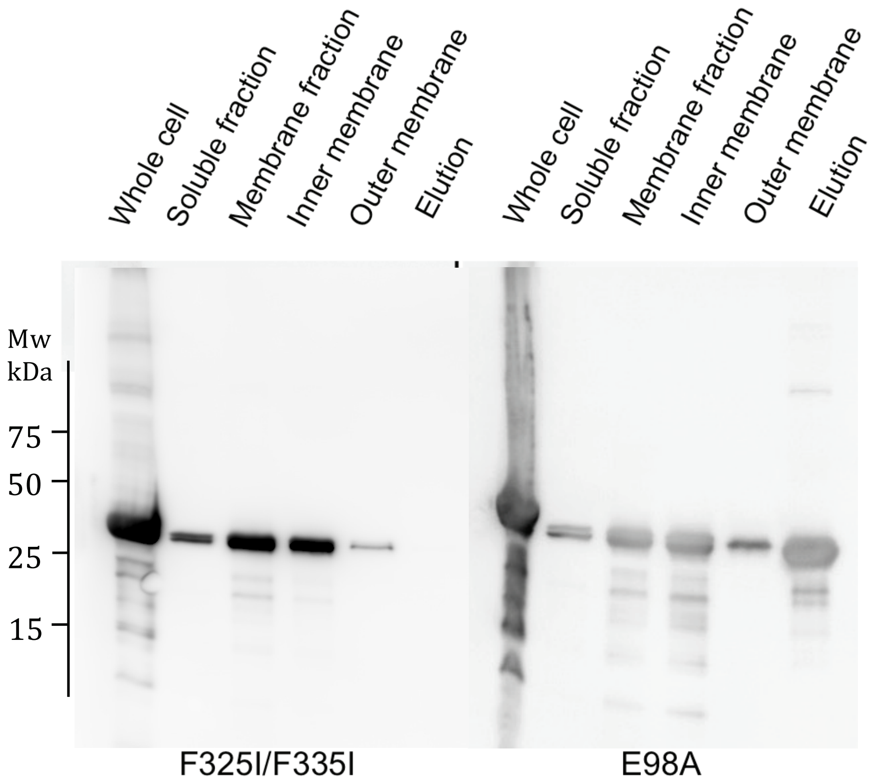

Supplementary Figure 5. Example of phenylalanine ladder mutant preparation. The F325I/F335I double mutant is not folded stably in the membrane and no protein is observed in the Ni-NTA elution fraction. Western blot analysis indicates low-level contamination of an unfolded species throughout purification stages until the Ni-NTA purification in which no protein is eluted. The right hand panel shows the same purification protocol for a successfully produced mutant (E98A) that was stably inserted and folded in the membrane.

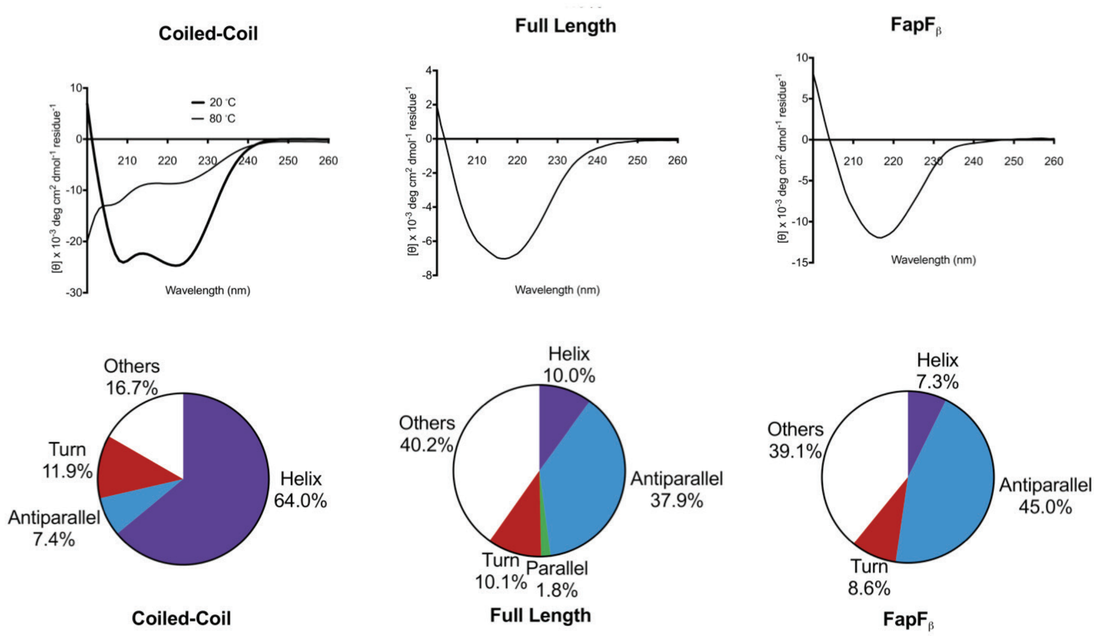

Supplementary Figure 6. Circular dichroism analyses of FapF coiled coil peptide D3-Q40, full-length FapF and $FapF_{ß}$. Based on these secondary structure content calculations, full-length FapF contains ~20-30 more residues with helical secondary structure than the truncated crystal construct, consistent with containing the D3-Q40 in a ~65 % alpha helical structure.

a

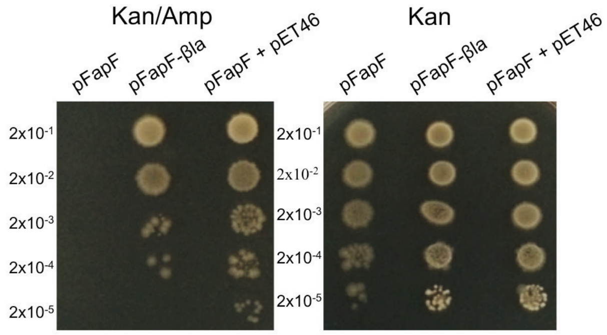

	$2x10^{-1}$	$2x10^{-2}$	$2x10^{-3}$	$2x10^{-4}$	$2x10^{-5}$
pFapF-βla	+	+	+-	+ -	-
pFapF	-	-	-	-	-
pFapF/pET46	+	+	+	+-	+ -

+ confluent growth   + - individual colonies   - no growth

b

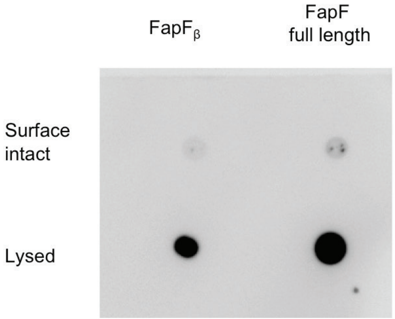

Supplementary Figure 7. Localisation of FapF N-terminus. (**a**) Ampicillin sensitivity assay. E. coli BL21 harbouring the indicated plasmid(s) were inoculated into LB and grown for 2 h before induction overnight at 21 ˚C with 0.5 mM IPTG. Cultures were serial diluted to the indicated values from OD_{600} 0.2 to $2x10^{-5}$. 5 μL of each dilution were plated on plates containing Amp/Kan and Kan and incubated overnight at 37 ˚C. The plates were then observed for the growth of colonies. On the control plate with kanamycin the cells grew to confluence at all dilutions. Representative plates show the growth of *E. coli* cells harbouring the indicated plasmids at the indicated dilutions on plates containing ampicillin and kanamycin as well as a kanamycin only control and results are summarised in tabular form. . (**b**) Surface antibody detection immunoblot. Intact bacteria were resuspended in PBS buffer to an OD_{600} of 1.5 and 2 μL dotted onto a nitrocellulose membrane. The same sample was diluted to an OD_{600} of 1.5 with SDS and boiled for 5 minutes to fully lyse the cells. The intact cells do not display surface binding of antihis HRP indicating that no histag is exposed on the extracellular surface. This is consistent with a periplasmic N-terminal domain for FapF.

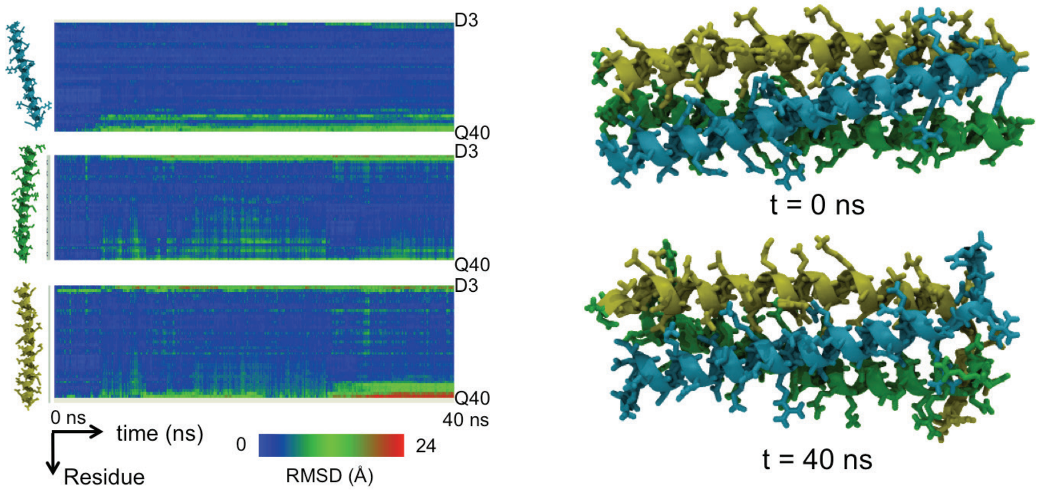

Supplementary Figure 8. Stability of parallel trimeric coiled coil built using CCBuilder V1.0. The peptide D3 to Q40 was used. Starting residue D3 was consensus ‘e’ register as found by multiple coiled coil prediction software. The core remained stable during short 40 ns of atomistic molecular dynamics simulation (right). The RMSD of all mainchain atoms of each peptide chain is shown (left). Each chain is shown separately coloured according to the figure on the right. The C-terminal region was least stable, which would be attached to the linker region in the full length protein.

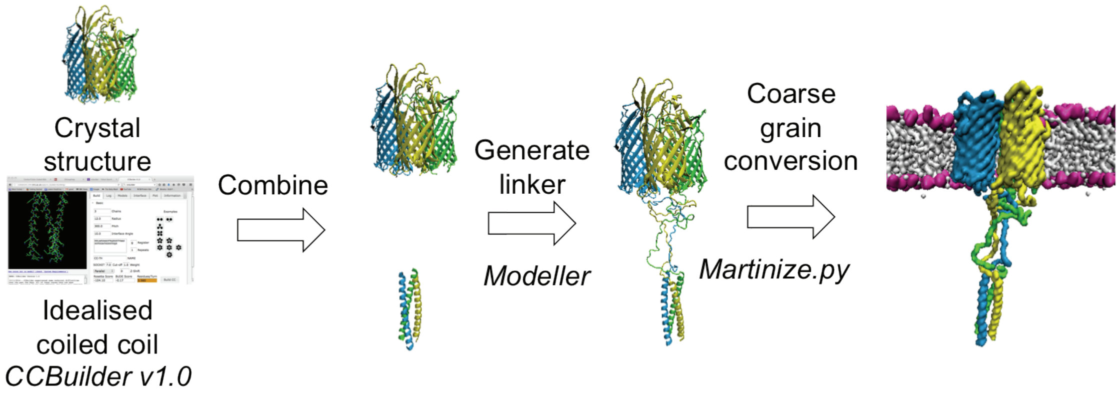

Supplementary Figure 9. Method for generating a model of full length FapF. The crystal structure presented in this work was combined with the idealised parallel trimeric coiled coil shown in Supplementary Figure 7. Modeller (www.salilab.org) was used to generate a linker with random coil secondary structure for residues 40 to 87. This was then converted into standard MARTINI v2.2 coarse grained representation for coarse grained molecular dynamics simulations in a POPE/POPG lipid membrane.

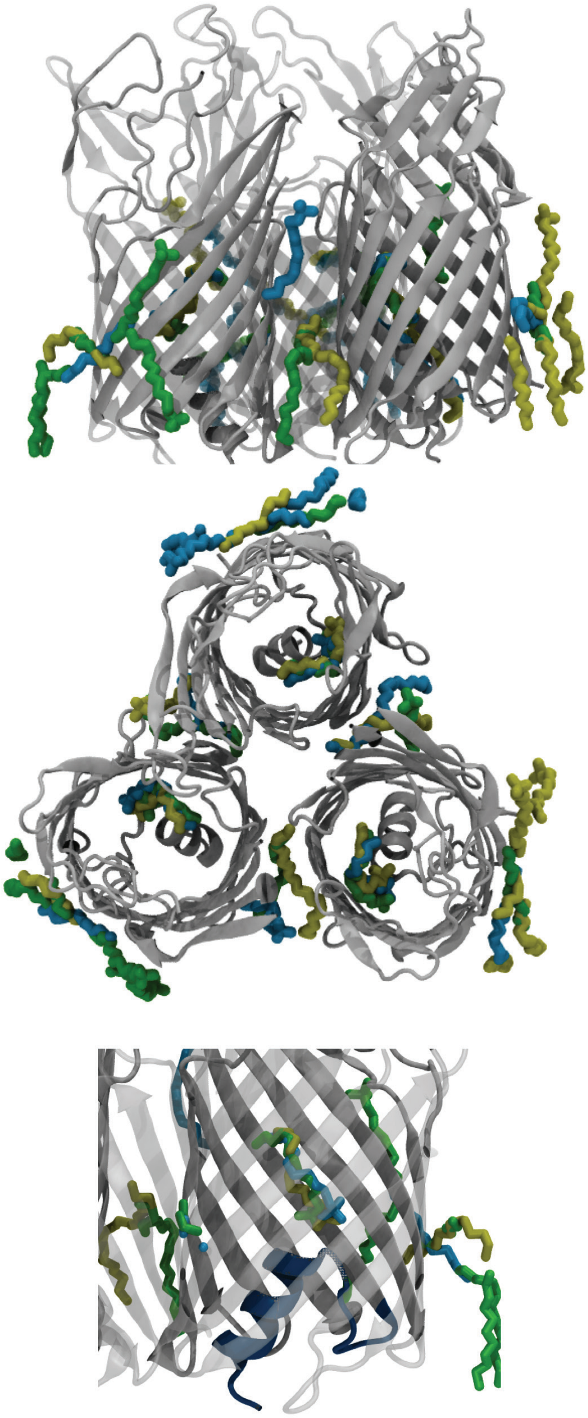

Supplementary Figure 10. Conservation of detergent binding sites amongst monomeric subunits. Here the detergent molcules are placed mapped onto each chain A (blue), B (yellow) and C (green). The only consistent binding site between all three chains is the detergent binding site above the helix plug (lower panel). All other observed binding sites were only resolved for 1 or 2 subunits.

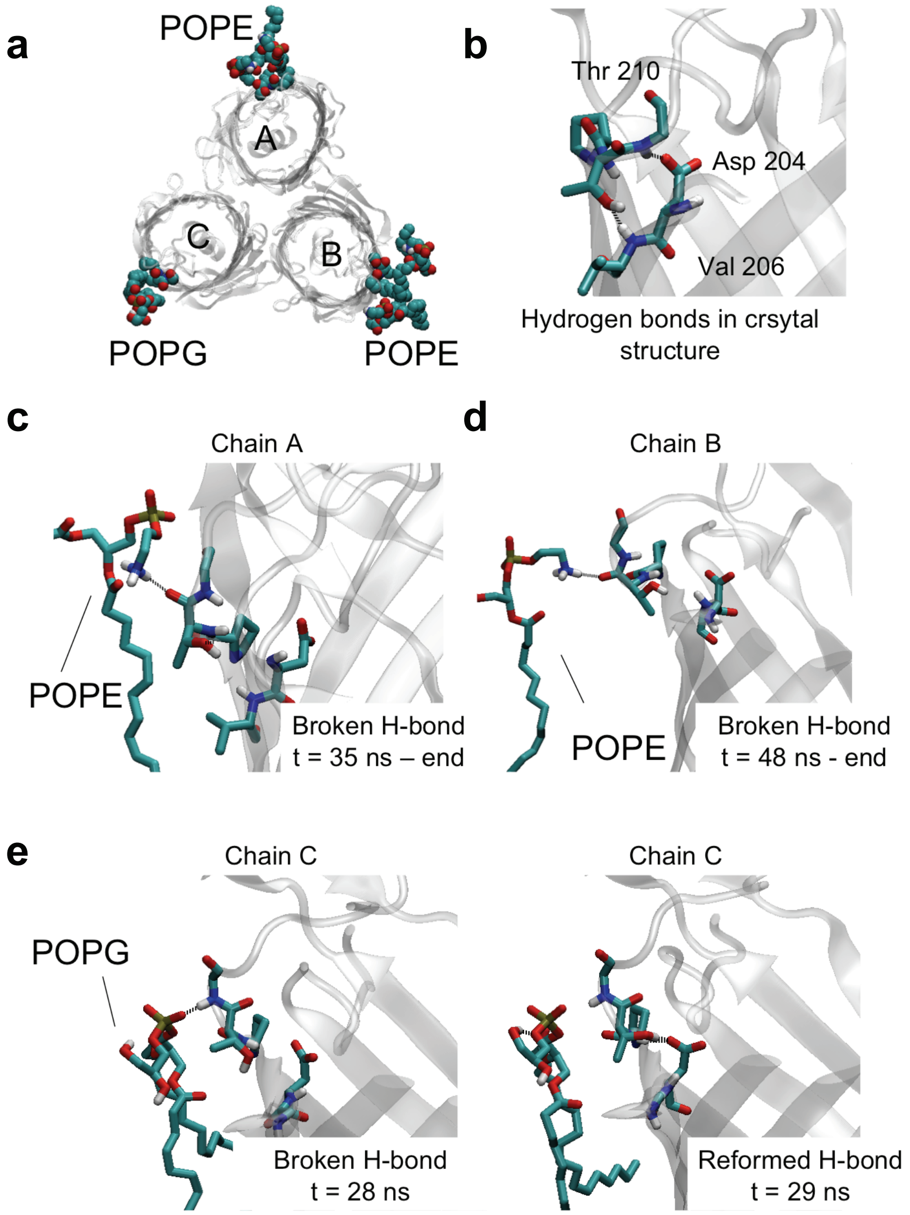

Supplementary Figure 11. Molecular dynamics analysis of interactions of lipids with the PTG motif in FapFß. . (**a**) POPE and POPG lipid molecules at the start of the simulation. . (**b**) The PTG motif and hydrogen bonds to conserved residues D204 and G203. (**c**) Presence of POPE displace the hydrogen bonds in chain A and this interaction with POPE remains throughout rest of simulation. (**d**) As in (**c**) but for chain B. (**e**) Interaction with POPG is reversible and native hydrogen bonds with D204 and G203 are observed to reform during simulation timescale.

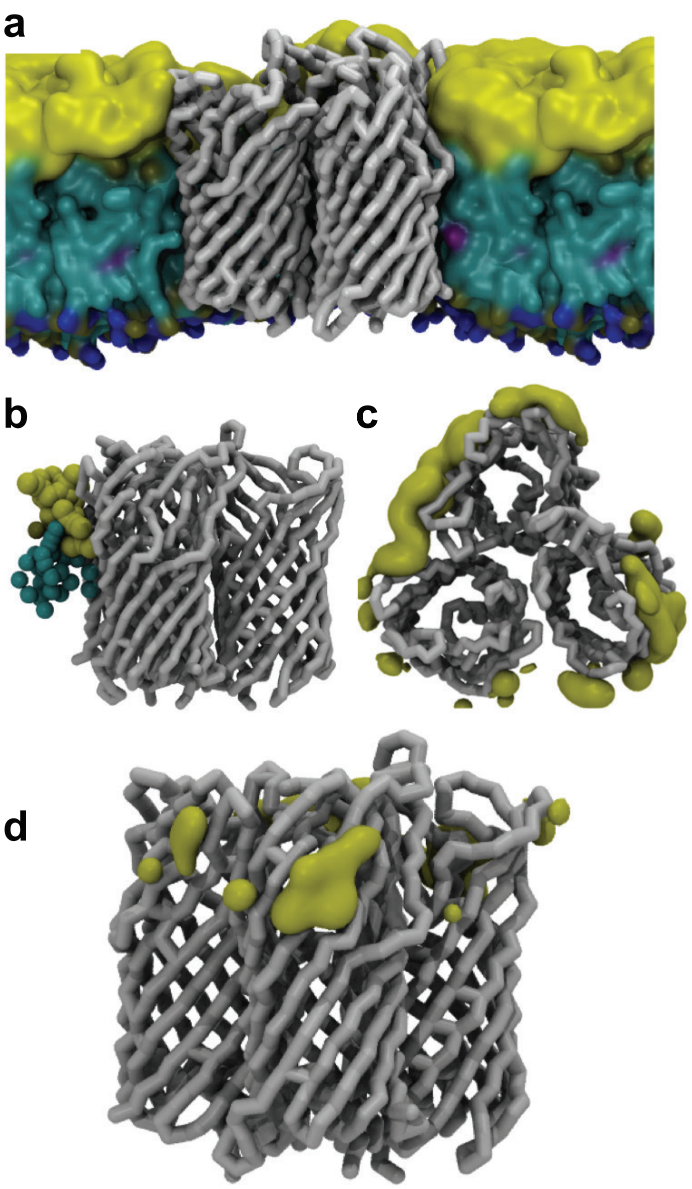

Supplementary Figure 12. Interactions of FapF$_{ß}$ extracellular loops with LPS. . (**a**) Starting point of a coarse-grained (CG) molecular dynamics simulation in which the initial configuration was generated by mapping the protein coordinates from an equilibrated POPE/POPG bilayer by alignment of phosphate groups of POPE/POPG and LPS. (**b**) A single LPS molecule binding to the interface of two subunits is shown. (**c**) Most occupied position of the LPS sugar moieties is shown as a yellow surface. (**d**). Side view of (**c**), highlighting a LPS sugar group binding in the region of the PTG motif discussed throughout this text.

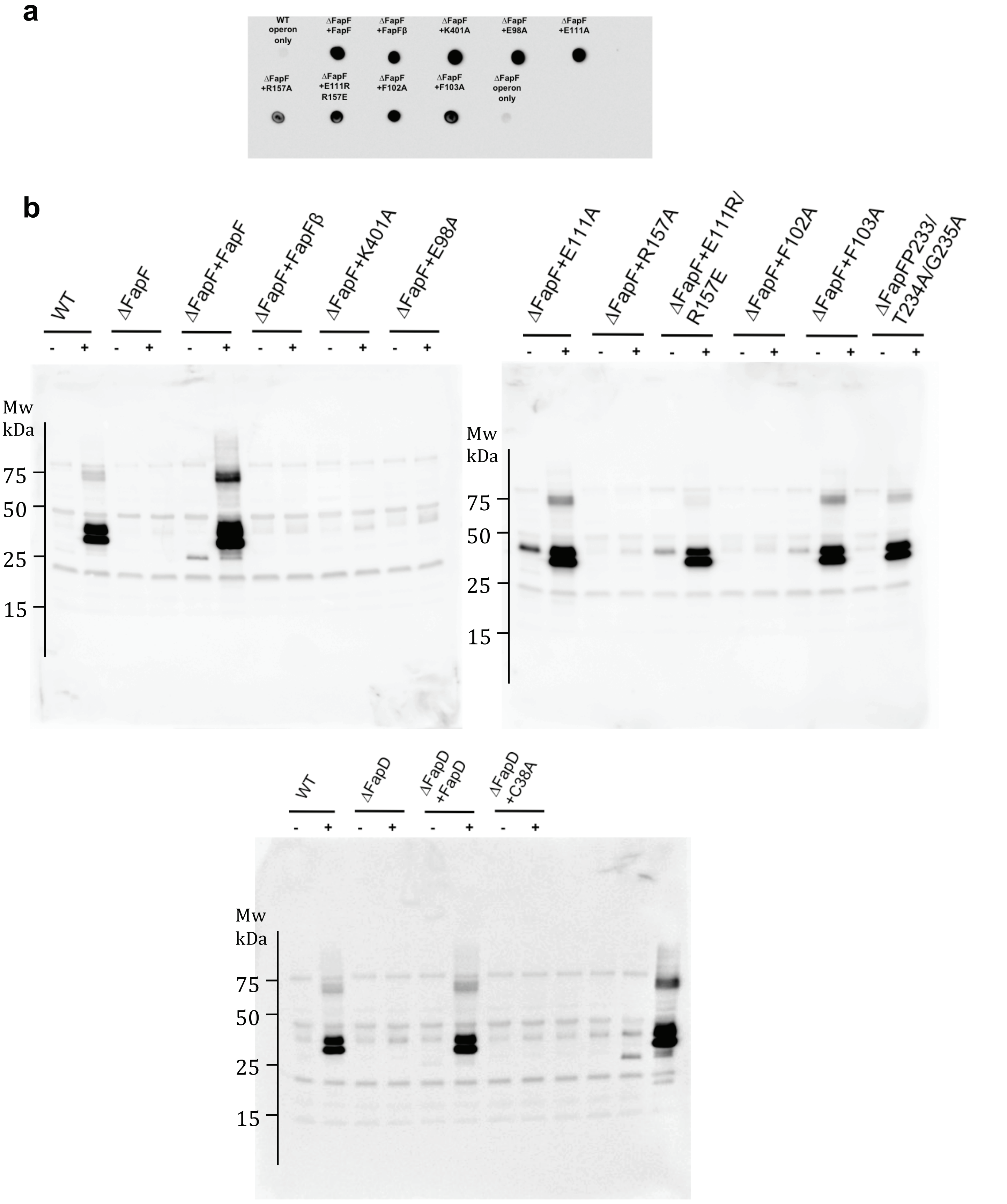

Supplementary Figure 13. (**a**) Expression of FapF in *E. coli* functional amyloid secretion assays. Water treated samples from strains used to test heterologous FapC amyloid production in *E. coli* (main text Figure 4) were dotted onto nitrocellulose to immunoblot for FapF production from the complementing plasmids, using an antibody against the histidine tag. (**b**) Full, uncropped gels for the amyloid secretion assays shown in Fig. 4 and Fig 5.

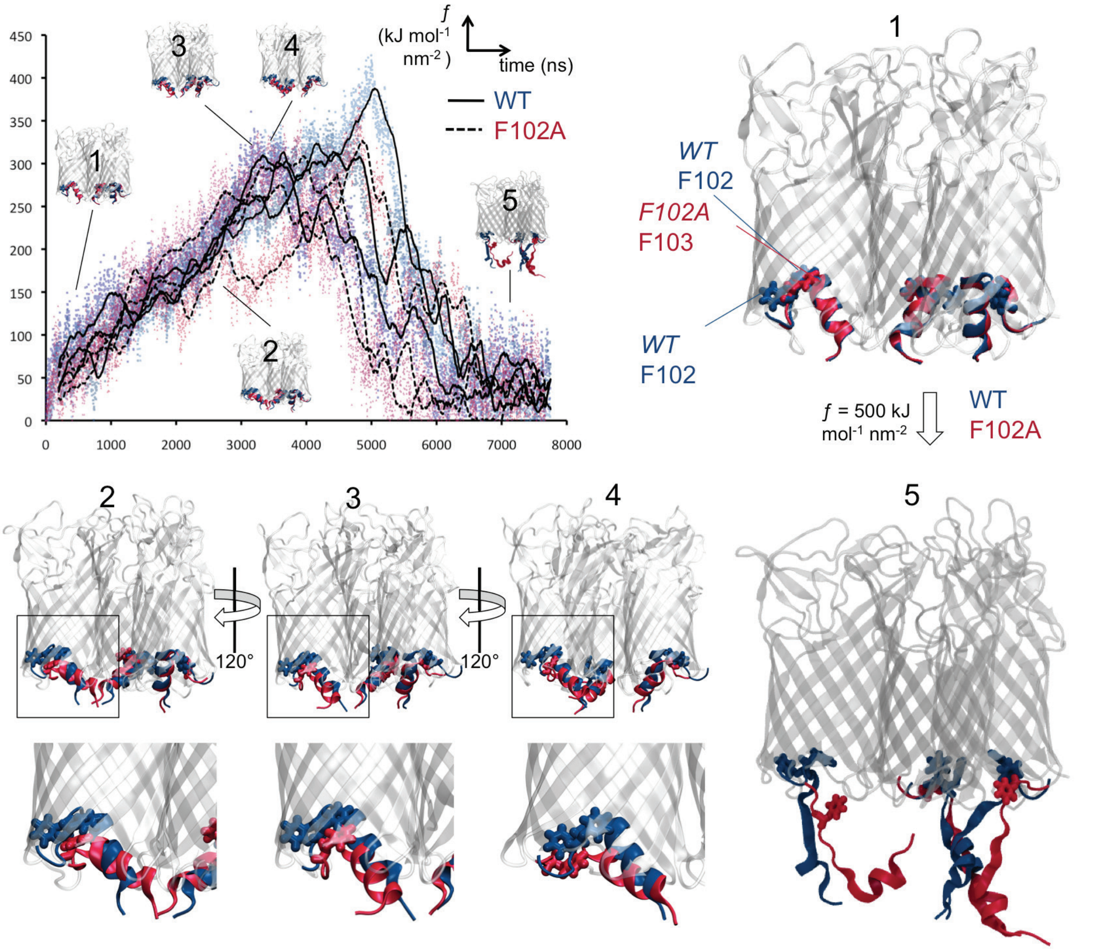

Supplementary Figure 14. Steered molecular dynamics (SMD) simulations in which the helix plug is pulled from the pore. An imaginary harmonic spring is applied to the centre of mass of each of the three helices for the wildtype (WT, blue) and the F102A mutant (red). A harmonic force is applied to the spring, inceasing proportional to the distance between the plug and the starting point. The plot indicates the force vs time for WT (solid line and blue data points) and F102A mutant (dashed line, red data points) applied to each helix. The snapshots 2, 3, and 4 correspond to the time each plug is removed for the F126A mutant and highlight the position of the F102 remaining within the barrel in the WT. F126 remains as an anchoring point in the WT and more force is required to remove the helix fully from the pore than for F102A. The final snapshot (5) demonstrates that F102 is still within the pore whilst the rest of the helix is unfolded for the WT. In the case of the F102A mutant the whole plug is removed from the pore. Taken together this data shows the F102 causes a barrier to plug removal and forms the main anchoring point of the helix plug within the barrel. The lines shown are a moving average of the data points over 0.1 ns intervals.

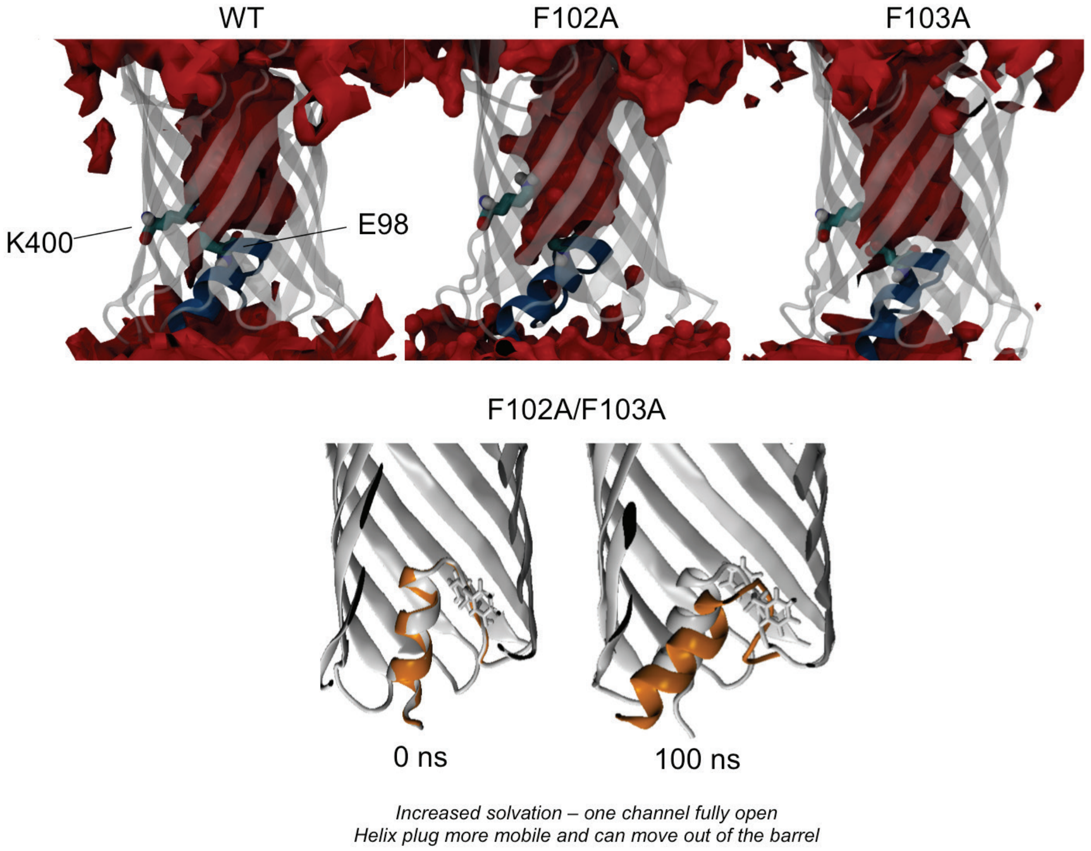

Supplementary Figure 15 Molecular dynamics analyses of plug stability of F102A/F103A single and double mutants. In the top panel the intact location of the ehlix plug in the single mutants F102A / F103A is indicated by the presence of the K400 E98 salt bridge as is also seen in the wildtype. The average solvation of each is shown as a red surface. In the lower panel, in one chain of the double mutant the helix plug is observed to move out from the barrel during the course of a 100 ns unbiased MD simulation.

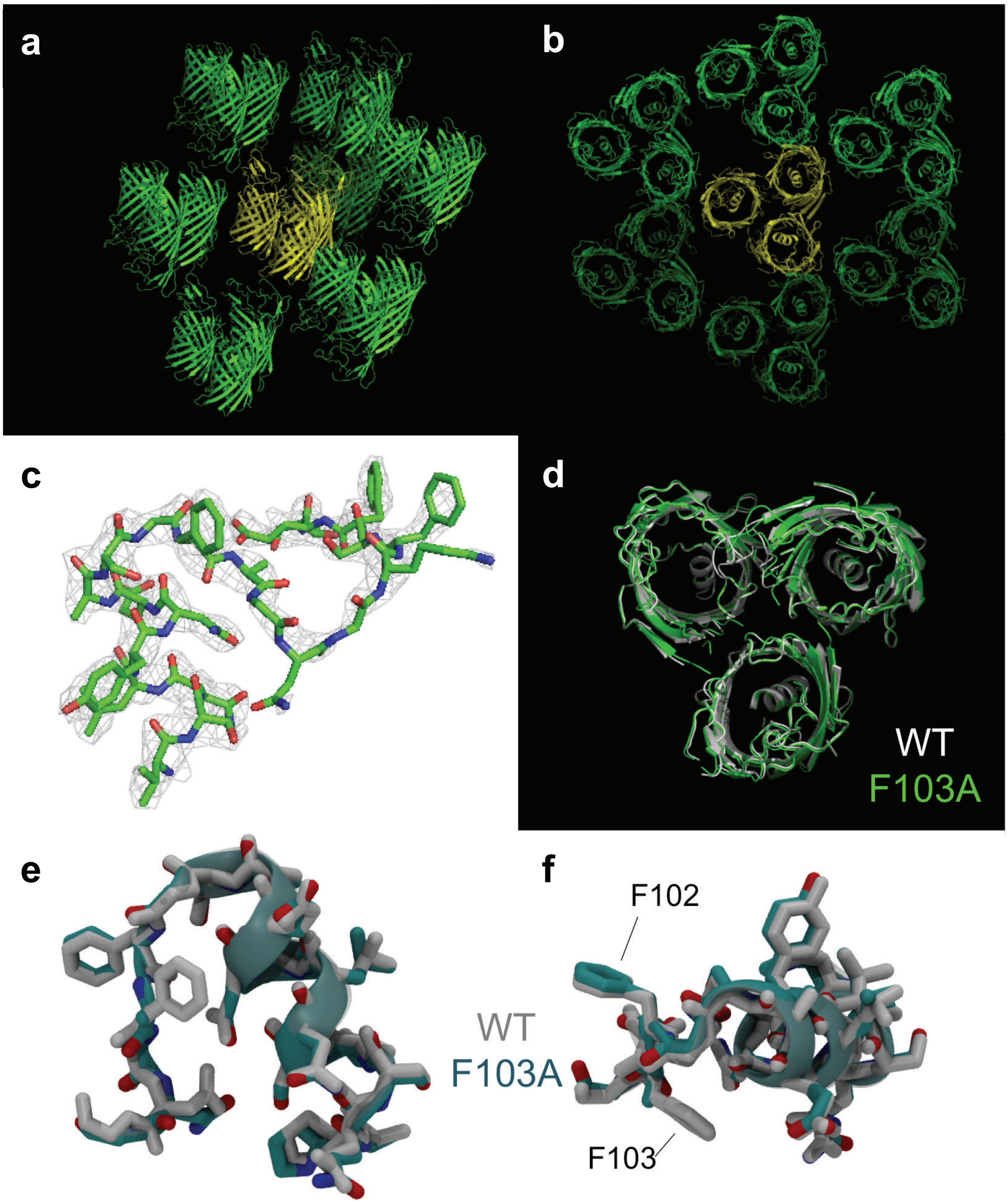

Supplementary Figure 16. Crystal strcucture of FapFß F103A mutant. (**a**) Sideview of the crystal packing in the asymmetric unit. One trimer is shown in yellow. (**b**) Top view of the crystal. (**c**) Example of X-ray density map. (**d**) Alignment of F103A (green) with the wildtype (white). (**e**) The helix plug position is maintained in the mutant compared to the wildtype. (**f**) Closer view of the sidechains in the helix plug.

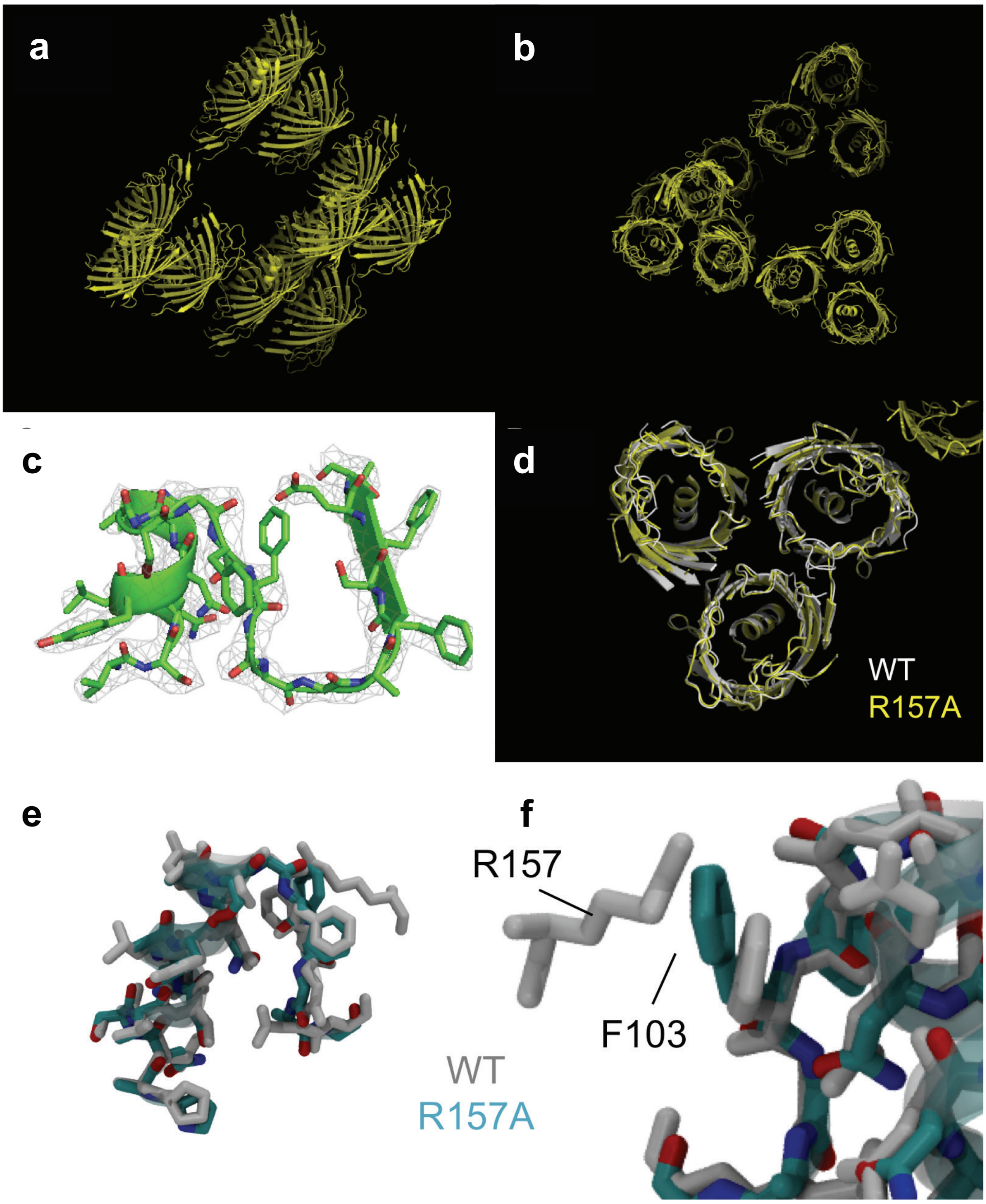

Supplementary Figure 17 Crystal structure of FapF$_{ß}$ R157A mutant. A. Sideview of the crystal packing in the asymmetric unit. B. Top view of the crystal. C. Example of X-ray density map. D. Alignment of R157A (yellow) with the wildtype (white). E. The helix plug position is maintained in the mutant compared to the wildtype. F. Closer view of the sidechains in the helix plug in which the position of F103 is seen to shift to occupy the void left by removal of R157 sidechain in the mutant.

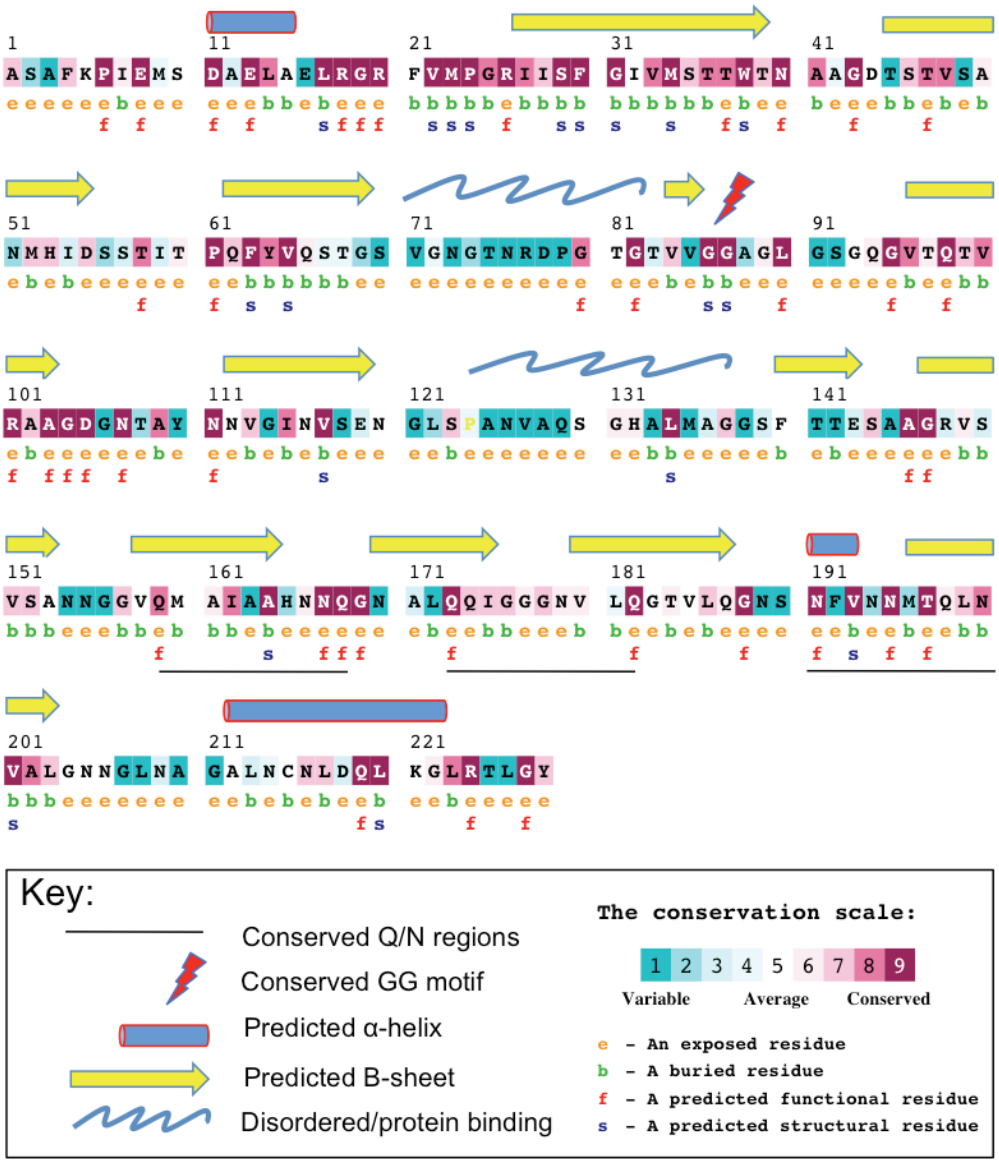

Supplementary Figure 18. Sequence analysis of UK4 FapE. Figure was generated using Consurf and PSIPRED outputs. A conserved GG motif, the putative target of FapD, at the end of the disordered region is highlighted. Conserved N- and Q- residues in the C-terminal region are underlined.

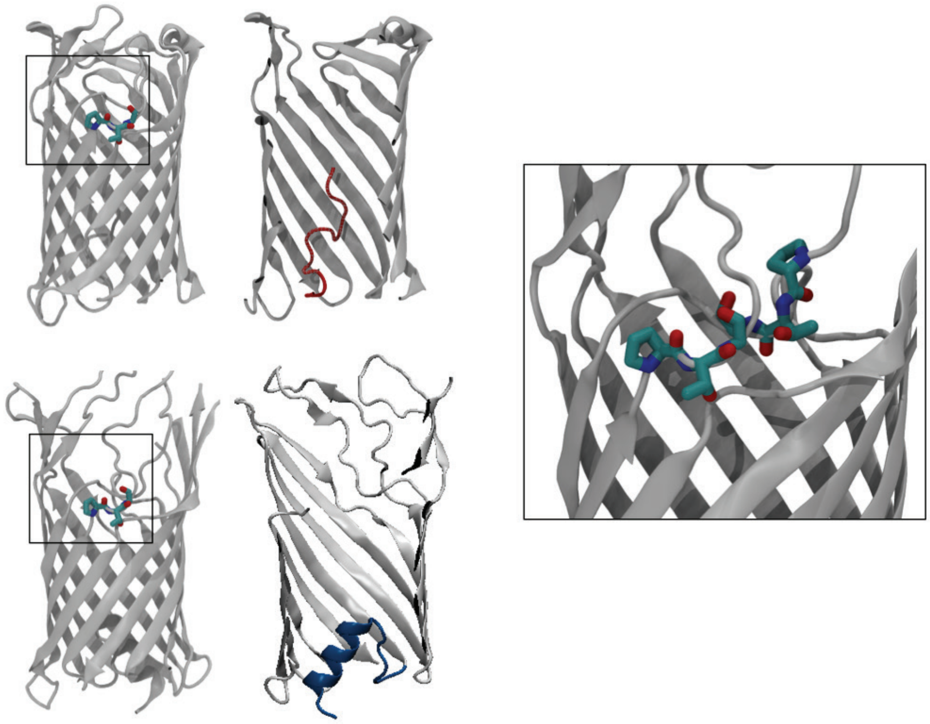

Supplementary Figure 19. Comparison of COG4313 family member Pput2275 with FapF$_{ß}$. The conserved PTG motif is shown in each. Top panel: Pput2275 is shown with the disordered N-termnus shown in red. Bottom: FapF$_{ß}$ is presented with the ordered helix plug shown in blue.

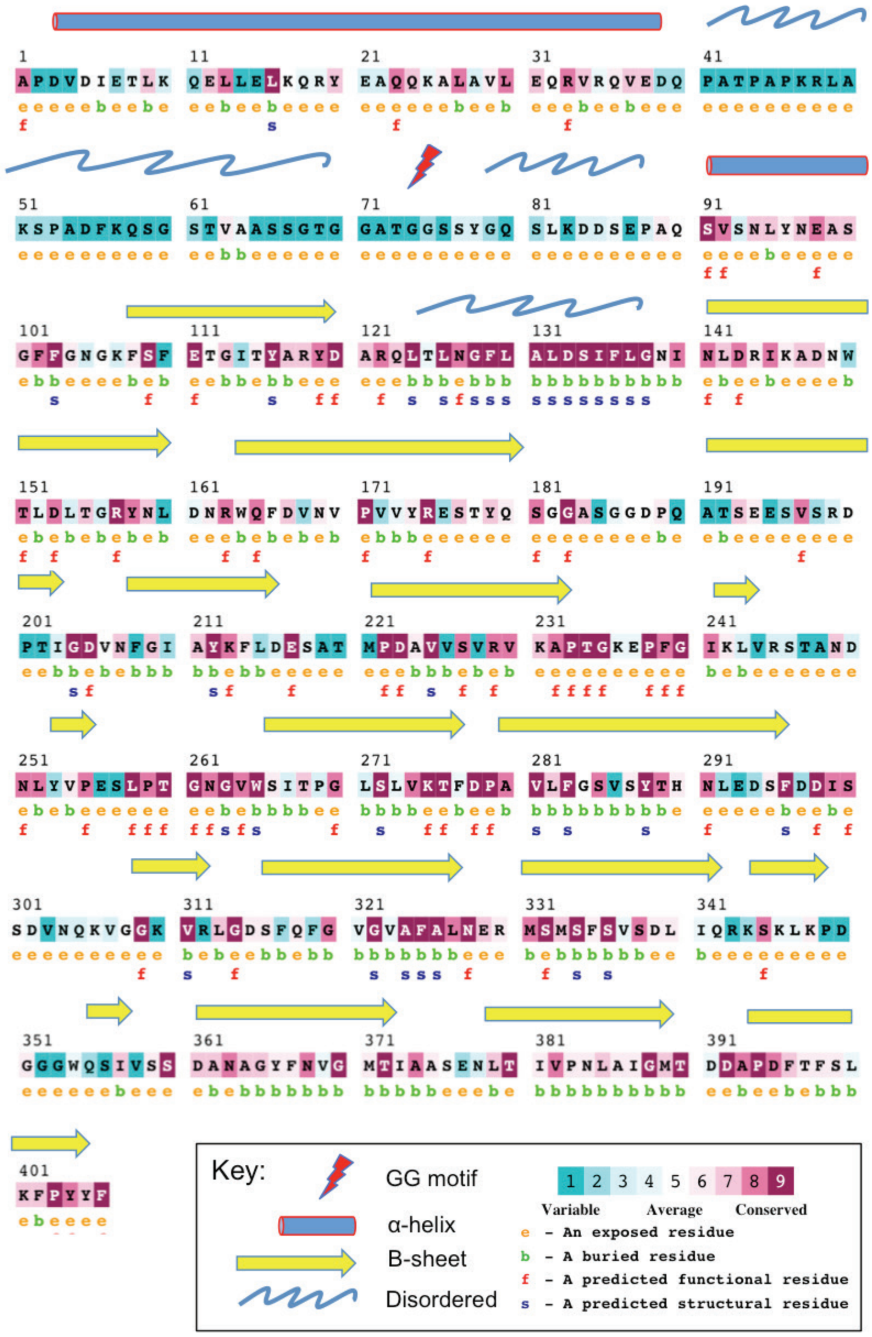

Supplementary Figure 20. Sequence analysis of UK4 FapF. Figure was generated using Consurf outputs combined with the secondary structures determined in this work.
